# Supplementary material for: Evolution of the WRKY66 Gene Family and Its Mutations Generated by the CRISPR/Cas9 System Increase the Sensitivity to Salt Stress in Arabidopsis
Source: Int J Mol Sci. 2023 Feb 4;24(4):3071. doi: 10.3390/ijms24043071 (PMC9959582; doi:10.3390/ijms24043071)
Supplement: Supplementary file 1 [file ijms-24-03071-s001.zip › Table S1.pdf]

Table S1. List of the sequences included in phylogenetic analyses for the *WRKY66* homologs. Species name, gene name, accession numbers, detailed genomic information, and classification are included.

Species	Name	Locus	No.of a.a	ORF	pI	Mw(kD)	Group
Abies alba	AalWRKY30_66	AALBA5B175278P1	445	1338	5.68	49.271	A
Amborella trichopoda	AtrWRKY30A	ATR0589G040	388	1167	7.91	43.198	B
	AtrWRKY30B	ATR0589G060	311	936	6.03	34.273	B
	AtrWRKY66	ATR1127G090	333	1002	5.77	37.208	C
Anthoceros_punctatus	ApuWRKY30_66	Apun_evm.model.utg000051l.449.1	761	2286	6.15	79.521	D
Arabidopsis thaliana	AtWRKY67	AT1G66550	249	750	6.04	28.363	C
	AtWRKY63	AT1G66600	241	726	5.63	27.379	C
	AtWRKY66	AT1G80590	235	708	5.8	26.403	C
	AtWRKY54	AT2G40750	346	1041	5.23	38.645	C
	AtWRKY46	AT2G46400	295	888	5.73	33.635	C
	AtWRKY70	AT3G56400	294	885	5.85	32.936	C
	AtWRKY41	AT4G11070	313	942	9.23	34.894	B
	AtWRKY53	AT4G23810	324	975	6.34	36.273	B
	AtWRKY62	AT5G01900	263	792	5.91	30.443	C
	AtWRKY38	AT5G22570	289	870	5.4	33.268	C
	AtWRKY30	AT5G24110	303	912	6.11	33.985	B
Azolla filiculoides	AfiWRKY30_66	Azfi_s0013.g013234	474	1425	5.78	52.371	D
Beta vulgaris	BvuWRKY30A	Bv1_003380_qcmg.t1	339	1020	6.42	37.844	B
	BvuWRKY30B	Bv3_052470_rpax.t1	361	1086	6.15	40.668	B
	BvuWRKY30C	Bv5_099120_wmfg.t1	368	1107	5.79	41.395	B
	BvuWRKY30D	Bv5_099120_wmfg.t2	325	978	5.45	36.364	B
	BvuWRKY66A	Bv4_089420_mdiu.t1	308	927	6.21	35.489	C

	BvuWRKY66B	Bv7_156860_hqwy.t1	327	984	5.96	37.392	C
Brachypodium distachyon	BdiWRKY30A	Bradi1g48770	363	1092	5.94	38.230	B
	BdiWRKY30B	Bradi2g15872	177	534	10.5	19.647	B
	BdiWRKY30C	Bradi2g15877	226	681	6.59	24.266	B
	BdiWRKY30D	Bradi2g45480	320	963	5.86	34.416	B
	BdiWRKY30E	Bradi3g34850	309	930	6.45	33.055	B
	BdiWRKY30F	Bradi4g28280	325	978	6.46	35.041	B
	BdiWRKY66A	Bradi1g17660	327	984	7.7	33.999	C
	BdiWRKY66B	Bradi4g44350	272	819	6.33	30.823	C
	BdiWRKY66C	Bradi1g63220	312	939	5.09	32.547	C
	BdiWRKY66D	Bradi1g63910	209	630	8.1	23.344	C
	BdiWRKY66E	Bradi4g25717	196	591	7.57	21.329	C
	BdiWRKY66F	Bradi2g53510	322	969	5.81	34.414	C
	BdiWRKY66G	Bradi2g22241	287	864	5.31	31.103	C
	BdiWRKY66H	Bradi2g30695	277	834	5.97	30.110	C
	BdiWRKY66I	Bradi2g53495	286	861	6.37	31.847	C
	BdiWRKY66J	Bradi2g44270	292	879	5.68	31.151	C
	BdiWRKY66K	Bradi2g53486	265	798	6.24	28.733	C
Coffea canephora	CcaWRKY30A	Cc02_g01860	377	1134	6.23	41.683	B
	CcaWRKY30B	Cc02_g39120	367	1104	5.26	40.795	B
	CcaWRKY30C	Cc08_g15910	342	1029	5.74	38.273	B
	CcaWRKY66	Cc02_g05270	333	1002	6.71	36.603	C
Fragaria vesca	FveWRKY30A	FVE13108	372	1119	6.64	40.958	B
	FveWRKY30B	FVE32076	355	1068	4.96	40.088	B
	FveWRKY66A	FVE21365	340	1023	6.43	37.927	C
	FveWRKY66B	FVE13546	447	1344	5.94	49.488	C
	FveWRKY66C	FVE13547	344	1035	5.41	38.757	C
	FveWRKY66D	FVE13549	231	696	9.32	25.955	C
	FveWRKY66E	FVE13552	300	903	4.93	33.880	C
	FveWRKY66F	FVE13554	297	894	7.17	33.544	C
Ginkgo biloba	GbiWRKY30_66A	Gb_00545	481	1446	5.38	53.127	A
	GbiWRKY30_66B	Gb_00546	140	423	4.32	15.536	A
	GbiWRKY30_66C	Gb_00547	579	1740	6.58	63.665	A

Glycine max	GmaWRKY30A	Glyma.01G224800	322	969	5.45	36.582	B
	GmaWRKY30B	Glyma.03G256700	362	1089	5.1	41.182	B
	GmaWRKY30C	Glyma.04G238300	364	1095	5.33	40.437	B
	GmaWRKY30D	Glyma.05G215900	363	1092	5.65	41.204	B
	GmaWRKY30E	Glyma.06G125600	364	1095	5.74	40.697	B
	GmaWRKY30F	Glyma.07G057400	369	1110	5.39	41.352	B
	GmaWRKY30G	Glyma.08G021900	359	1080	5.96	40.620	B
	GmaWRKY30H	Glyma.16G026400	373	1122	5.1	41.456	B
	GmaWRKY30I	Glyma.19G254800	362	1089	5.73	41.243	B
	GmaWRKY66A	Glyma.18G213200	299	900	6.03	33.869	C
	GmaWRKY66B	Glyma.03G002300	235	708	4.81	26.758	C
	GmaWRKY66C	Glyma.16G219800	265	798	5.8	30.543	C
	GmaWRKY66D	Glyma.14G186100	240	723	9.05	26.970	C
	GmaWRKY66E	Glyma.04G223300	317	954	5.46	35.856	C
	GmaWRKY66F	Glyma.14G186000	303	912	6.83	33.920	C
	GmaWRKY66G	Glyma.14G185800	329	990	6.1	37.387	C
	GmaWRKY66H	Glyma.13G267700	270	813	5.84	30.601	C
	GmaWRKY66I	Glyma.06G142000	319	960	5.45	35.954	C
	GmaWRKY66J	Glyma.13G267600	300	903	6.09	34.101	C
	GmaWRKY66K	Glyma.13G267500	295	888	5.93	33.865	C
	GmaWRKY66L	Glyma.13G267400	294	885	6.13	33.766	C

	GmaWRKY66M	Glyma.09G274000	300	903	5.95	34.224	C
Gnetum montanum	GmoWRKY30_66	TnS000068155t02	375	1128	5.26	41.441	A
Marchantia polymorpha	MpoWRKY30_66	Mapoly0036s0136.1	652	1959	5.66	70.358	D
Nelumbo nucifera	NnuWRKY30A	NNU_01465	362	1089	6.23	40.544	B
	NnuWRKY30B	NNU_02487	363	1092	5.27	40.360	B
	NnuWRKY30C	NNU_02488	362	1089	5.51	40.549	B
	NnuWRKY66A	NNU_12194	329	990	5.46	37.057	C
	NnuWRKY66B	NNU_24385	326	981	6.13	36.496	C
Oryza sativa	OsaWRKY30A	LOC_Os01g46800	374	1125	7.02	29.056	B
	OsaWRKY30B	LOC_Os05g49620	277	834	5.53	29.872	B
	OsaWRKY30C	LOC_Os06g06360	391	1176	5.79	41.403	B
	OsaWRKY30D	LOC_Os08g29660	319	960	6.18	33.550	B
	OsaWRKY30E	LOC_Os09g16510	361	1086	5.96	37.944	B
	OsaWRKY30F	LOC_Os11g45920	399	1200	8.89	44.808	B
	OsaWRKY66A	LOC_Os12g02440	192	579	5.7	22.059	C
	OsaWRKY66B	LOC_Os01g60490	265	798	6.63	29.807	C
	OsaWRKY66C	LOC_Os01g60520	283	852	5.86	31.037	C
	OsaWRKY66D	LOC_Os01g60600	358	1077	5.32	37.867	C
	OsaWRKY66E	LOC_Os03g20550	210	633	8.38	23.642	C
	OsaWRKY66F	LOC_Os03g21710	356	1071	5.15	36.384	C
	OsaWRKY66G	LOC_Os05g25770	326	981	5.53	33.988	C
	OsaWRKY66H	LOC_Os05g40060	331	996	4.61	34.901	C

	OsaWRKY66I	LOC_Os05g40080	328	987	4.88	34.720	C
	OsaWRKY66J	LOC_Os12g02400	337	1014	5.5	37.542	C
	OsaWRKY66K	LOC_Os12g02420	222	669	8.73	25.077	C
	OsaWRKY66L	LOC_Os07g48260	333	1002	6.14	34.838	C
	OsaWRKY66M	LOC_Os11g02520	280	843	5.82	31.662	C
	OsaWRKY66N	LOC_Os11g02480	224	675	8.54	25.188	C
	OsaWRKY66O	LOC_Os10g18099	276	831	7.22	30.653	C
	OsaWRKY66P	LOC_Os11g02470	335	1008	5.46	37.202	C
Picea abies	PabWRKY30_66	PAB00048758	572	1719	7.03	63.238	A
Pinus pinaster	PpiWRKY30_66A	PPI00017232	149	450	4.39	16.636	A
	PpiWRKY30_66B	PPI00033471	123	372	5.55	14.042	A
Pinus sylvestris	PsyWRKY30_66A	PSY00032986	119	360	9.78	12.971	A
	PsyWRKY30_66B	PSY00034387	246	741	5.47	26.882	A
Pinus taeda	PtaWRKY30_66	PTA00045106	477	1434	5.56	52.530	A
Pseudotsuga menziesii	PmeWRKY30_66	PME00010021	472	1419	5.61	52.498	A
Populus trichocarpa	PtrWRKY30A	Potri.001G092900	338	1017	5.79	37.967	B
	PtrWRKY30B	Potri.002G168700	363	1092	5.82	40.939	B
	PtrWRKY30C	Potri.003G138600	342	1029	5.25	38.391	B
	PtrWRKY30D	Potri.012G031700	371	1116	5.56	41.360	B

	PtrWRKY30E	Potri.014G096200	365	1098	5.1	41.180	B
	PtrWRKY66A	Potri.006G109100	333	1002	6.04	37.688	C
	PtrWRKY66B	Potri.013G090300	324	975	5.3	36.692	C
	PtrWRKY66C	Potri.016G137900	321	966	5.83	36.045	C
Selaginella moellendorffii	SmoWRKY30_66A	gene1467	512	1539	6.03	54.235	D
	SmoWRKY30_66B	gene18729	404	1215	8.09	44.109	D
	SmoWRKY30_66C	gene22742	512	1539	6.17	54.190	D
	SmoWRKY30_66D	gene5840	329	990	6.04	35.566	D
Sequoiadendron giganteum	SgiWRKY30_66A	SEGI_21579	494	1485	5.69	54.029	A
	SgiWRKY30_66B	SEGI_34413	444	1335	5.13	49.021	A
Solanum lycopersicum	SlyWRKY30A	Solyc01g095630	336	1011	5.55	37.727	B
	SlyWRKY30B	Solyc03g007380	353	1062	6.09	40.063	B
	SlyWRKY30C	Solyc05g050300	195	588	6.06	22.573	B
	SlyWRKY30D	Solyc05g050330	244	735	6.02	27.538	B
	SlyWRKY30E	Solyc05g050340	327	984	5.79	36.725	B
	SlyWRKY30F	Solyc08g008280	360	1083	5.51	40.709	B
	SlyWRKY30G	Solyc08g082110	339	1020	5.41	38.574	B
	SlyWRKY30H	Solyc10g009550	290	873	5.45	33.655	B
	SlyWRKY66A	Solyc03g095770	273	822	6.02	31.533	C
	SlyWRKY66B	Solyc09g015770	291	876	5.51	33.250	C

Taxus baccata	TbaWRKY30_66	TBA00021329	496	1491	5.79	54.390	A
Thuja plicata	TplWRKY30_66A	Thupl.29379665s0021	501	1506	5.46	54.962	A
	TplWRKY30_66B	Thupl.29380446s0004	439	1320	4.91	48.375	A
Vitis vinifera	VviWRKY30A	GSVIVT01019511001	342	1029	6.05	38.562	B
	VviWRKY30B	GSVIVT01027069001	360	1083	5.16	40.234	B
	VviWRKY30C	GSVIVT01028718001	364	1095	5.45	40.043	B
	VviWRKY66A	GSVIVT01030174001	331	996	5.76	37.485	C
	VviWRKY66B	GSVIVT01032661001	288	867	5.71	32.693	C
Welwitschia mirabilis	WmiWRKY30_66	W.mirabilis.10165	500	1503	5.66	55.601	A
