# Supplementary material for: Evolution of the WRKY66 Gene Family and Its Mutations Generated by the CRISPR/Cas9 System Increase the Sensitivity to Salt Stress in Arabidopsis
Source: Int J Mol Sci. 2023 Feb 4;24(4):3071. doi: 10.3390/ijms24043071 (PMC9959582; doi:10.3390/ijms24043071)
Supplement: Supplementary file 1 [file ijms-24-03071-s001.zip › Table S2.pdf]

Table S2 Table showing average amino acid composition of plant *WRKY66* homologs.

Amino Acids	Average amino acid composition of WRKY66s
Ala	6.42
Cys	2.01
Asp	6.10
Glu	6.11
Phe	3.91
Gly	6.32
His	3.10
Ile	3.91
Lys	5.03
Leu	7.01
Met	2.36
Asn	4.69
Pro	6.36
Gln	5.25
Arg	5.07
Ser	11.76
Thr	6.16
Val	4.50
Trp	0.97
Tyr	2.96
