# Supplementary material for: Evolution of the WRKY66 Gene Family and Its Mutations Generated by the CRISPR/Cas9 System Increase the Sensitivity to Salt Stress in Arabidopsis
Source: Int J Mol Sci. 2023 Feb 4;24(4):3071. doi: 10.3390/ijms24043071 (PMC9959582; doi:10.3390/ijms24043071)
Supplement: Supplementary file 1 [file ijms-24-03071-s001.zip › Table S3.pdf]

Table S3. Analysis of *cis*-acting elements of promoters. The amounts are the sum of c*is*-elements in sense and antisense strand.

Name of elements	Amount	Sequence	Function
ABRE	1	ACGTG	cis-acting element involved in the abscisic acid responsiveness
CGTCA-motif	1	CGTCA	cis-acting regulatory element involved in the MeJA-responsiveness
G-Box	1	CACGTT	cis-acting regulatory element involved in light responsiveness
GARE-motif	2	TCTGTTG	gibberellin-responsive element
I-box	2	GATAAGGTG	part of a light responsive element
TGACG-motif	1	TGACG	cis-acting regulatory element involved in the MeJA-responsiveness
W box	1	TTGACC	/
MYB	2	CAACAG	/
MYC	1	CATTTG	/
GT1-motif	1	GGTTAAT	light responsive element
