# Supplementary material for: Evolution of the WRKY66 Gene Family and Its Mutations Generated by the CRISPR/Cas9 System Increase the Sensitivity to Salt Stress in Arabidopsis
Source: Int J Mol Sci. 2023 Feb 4;24(4):3071. doi: 10.3390/ijms24043071 (PMC9959582; doi:10.3390/ijms24043071)
Supplement: Supplementary file 1 [file ijms-24-03071-s001.zip › Table S5.pdf]

Table S5 Changes in the expression of genes in ABA-mediated signaling pathway in the WT and *wrky66* plants after the NaCl or ABA treatments.

Gene locus	Gene name	WTNa_vs _WTCK	MUNa_vs _MUCK	WTABA_vs _WTCK	MUABA_vs _MUCK
AT4G08500	MEKK1	-0.342296465	0.335407092	-0.062020471	0.104338506
AT4G29810	MKK2	0.659995745	0.824461244	0.593473447	0.068431421
AT4G01370	MPK4	0.540243748	0.446427192	0.196733897	-0.559976489
AT2G43790	MPK6	0.496647943	0.669792663	0.242087644	-0.217273262
AT4G17870	PYR1	-0.516189516	-1.006732406	-2.348933122	-3.349618938
AT2G26040	PYL2	0.122394994	0.231024874	-3.633070508	-2.660318308
AT2G38310	PYL4	-0.083637238	-0.277458359	-4.235616998	-4.715397946
AT5G05440	PYL5	0.448774476	-0.005348444	-3.491111538	-3.956231093
AT2G40330	PYL6	-0.047687538	-0.810703123	-8.669761884	-11.38735531
AT4G01026	PYL7	2.373349764	2.484085874	0.99360138	0.630283846
AT5G53160	PYL8	1.027607128	0.902510066	-0.823028616	-1.44845205
AT4G26080	ABI1	-0.400289699	-0.287682139	3.067250747	3.160460286
AT5G57050	ABI2	-0.720840321	-0.793827122	4.962641821	4.326615538
AT5G51760	AHG1	12.45422817	2.054288653	15.29603289	6.733083467
AT1G72770	HAB1	0.704830239	0.488163328	4.106374032	2.389023652
AT1G17550	HAB2	0.053179065	0.230477338	2.096805142	1.512941746
AT5G59220	HAI1	1.030021987	2.493124215	7.68526705	8.575186075
AT1G07430	HAI2	0.459581754	-0.335147593	8.303001491	7.233723994
AT2G29380	HAI3	8.070747095	0.320437942	17.72582826	9.562041456
AT3G11410	PP2CA	-0.892062169	-1.347485591	4.582142415	4.244251445
AT5G08590	SnRK2.1	-0.112142427	-0.78722252	1.541828591	0.434870876
AT3G50500	SnRK2.2	1.159176981	0.779646306	1.259710932	-0.244621383
AT5G63650	SnRK2.5	1.052426998	0.809188521	-0.681916125	-1.496781452
AT4G33950	SnRK2.6	1.862138952	1.816287227	2.262918587	1.512838721
AT4G40010	SnRK2.7	-1.806326171	-1.687769844	5.612426676	6.028809991
AT1G78290	SnRK2.8	1.378458507	1.321235786	-0.175401826	-0.830299073
AT2G23030	SnRK2.9	-0.278921089	-0.481664091	1.339677059	0.526723338
AT1G60940	SnRK2.10	0.403624004	0.101103856	1.030424706	0.027789528
AT4G01250	WRKY22	1.618816691	1.536154574	0.909987396	-0.303121294
AT2G30250	WRKY25	1.999528228	1.645837524	0.570478156	-1.338278909
AT4G23550	WRKY29	1.507616997	2.432515222	-2.22237027	-2.054333833
AT2G38470	WRKY33	2.071714447	2.253871326	0.721416067	-1.069193074
AT1G20630	CAT1	1.442311193	1.771151339	1.320238981	1.168490684
AT4G35090	CAT2	1.624902315	1.950444788	0.157816893	1.225223213
AT1G20620	CAT3	1.511537576	1.446706612	-0.229945152	-0.060865306
