# Supplementary material for: Evolution of the WRKY66 Gene Family and Its Mutations Generated by the CRISPR/Cas9 System Increase the Sensitivity to Salt Stress in Arabidopsis
Source: Int J Mol Sci. 2023 Feb 4;24(4):3071. doi: 10.3390/ijms24043071 (PMC9959582; doi:10.3390/ijms24043071)
Supplement: Supplementary file 1 [file ijms-24-03071-s001.zip › Table S6.pdf]

Table S6. The primer list of all genes used in this study.

Gene name	Gene ID	Forward primer (5′→3′)	Reverse primer (5′→3′)	Amplicon length (bp)	Function
W66-sgRNA	AT1G80590	tgattGTCCCAAGAAATT ACCATGG	aaacCCATGGTAATTTCT TGGGACA	19	For CRISPR vector construction
JD:AtWRKY66	AT1G80590	TTGAGATTGATGCGAA GGC	ACGACGAAGAAATACA TCACTGA	1082	For CRISPR lines detection
W66-GFP	AT1G80590	CGGGATCCATGTCTCT TGAGATTGATGCGA	GCTCTAGAAGATTTATT AATGTTCAATCCT	708	For subcellular localization analysis
W66-pGBKT7	AT1G80590	GGAATTCCATATGTCTC TTGAGATTGATGCG	CGGAATTCTTAAGATT TATTAATGTTCAAT	708	For transcription activation assay
AtWRKY66-qPCR	AT1G80590	ACCATGGAGGATAAAG ACACA	GCAGCAACGTTCTCCA AGTAC	111	For qRT-PCR
BGLU24	AT5G28510	ACAACCTCACACGTCA TGAGAA	GGATGGACGAACTCCC TCAC	74	For qRT-PCR
RD20	AT2G33380	TAGCAAATACGCGCTA ACGG	TGCCATTCGATTTCCCT CGG	79	For qRT-PCR
ABA1	AT5G67030	AATTTTCCCGCGCGGT TTAG	CTTCCTCGTCGATTTC GGAGT	108	For qRT-PCR
MYB102	AT4G21440	CGGTCTTAAACACGCC ATCCT	CTCAGTGCTGCAACTG CTACT	83	For qRT-PCR
SnRK2-6	AT4G33950	CTCCTGCAGGCACTCA GAAT	CTCTCCGCTACTGTCG ATGTC	119	For qRT-PCR
WRKY22	AT4G01250	CGGATACGGTGGTTAG CGAT	CCGAGTTCCCGGAAA AGCTA	78	For qRT-PCR
HAI3	AT2G29380	TGCTCTGTAGCGCGTA TGTG	ACTGACGCTTCCGTAC AAGC	95	For qRT-PCR
AHG1	AT5G51760	CGAAGAAACTCCCTCG AGCC	CAACGTTGCAGCCAAT ACACT	100	For qRT-PCR
PYL6	AT2G40330	CGGCGGGTAACGATAA GGAA	GCGAGTTTAGCCAGCG ATTG	82	For qRT-PCR
PYL8	AT5G53160	TCTCTCTTCACCCCGA	TCCTTTGTGTTTCCTTC	91	For qRT-PCR

		GACT	TGGT		
AtActin	AT3G18780	TGTCGCCATCCAAGCTGTTCTCT	GTGAGACACACCATCACCAGAAT	85	For qRT-PCR
