# Supplementary figures and images for: Evolution of the WRKY66 Gene Family and Its Mutations Generated by the CRISPR/Cas9 System Increase the Sensitivity to Salt Stress in Arabidopsis
Source: Int J Mol Sci. 2023 Feb 4;24(4):3071. doi: 10.3390/ijms24043071 (PMC9959582; doi:10.3390/ijms24043071)

(a)

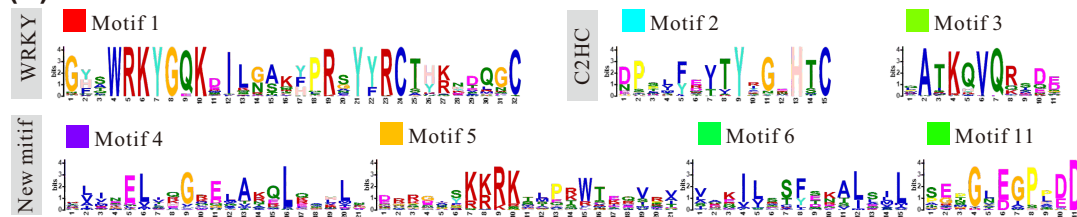

(b)

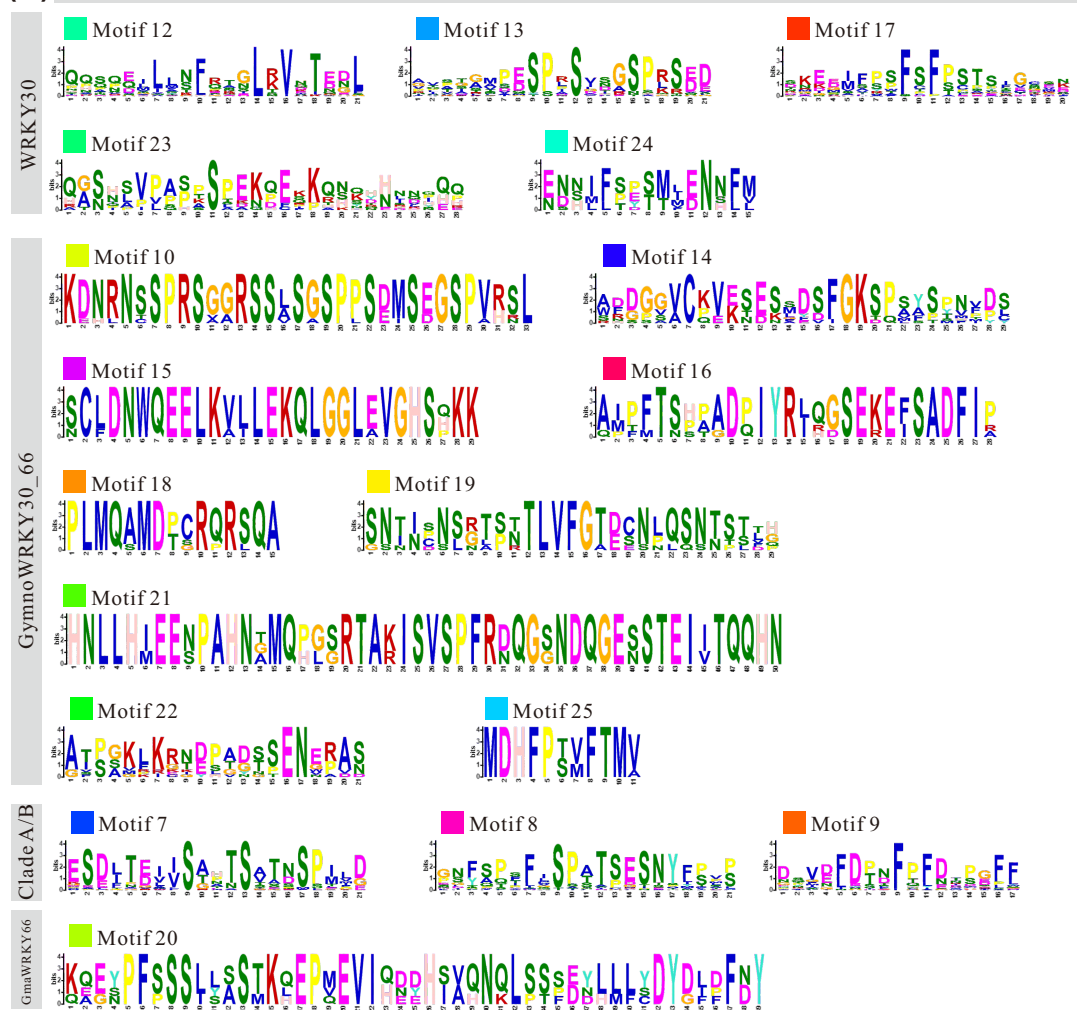

Supplement: Supplementary file 1 [file ijms-24-03071-s001.zip › Fig.S1.pdf]

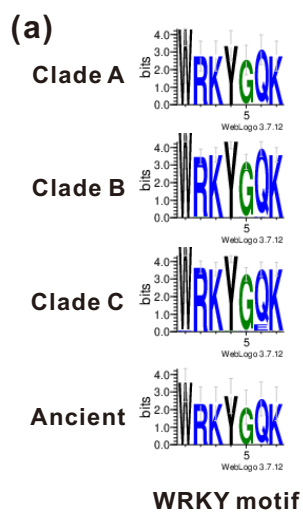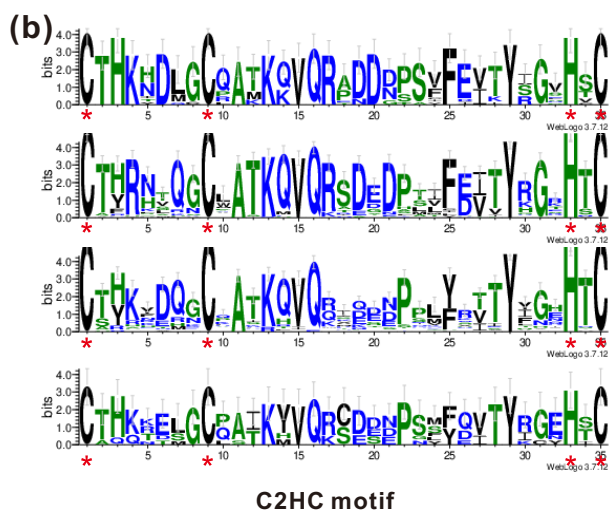

Supplement: Supplementary file 1 [file ijms-24-03071-s001.zip › Fig.S2.pdf]

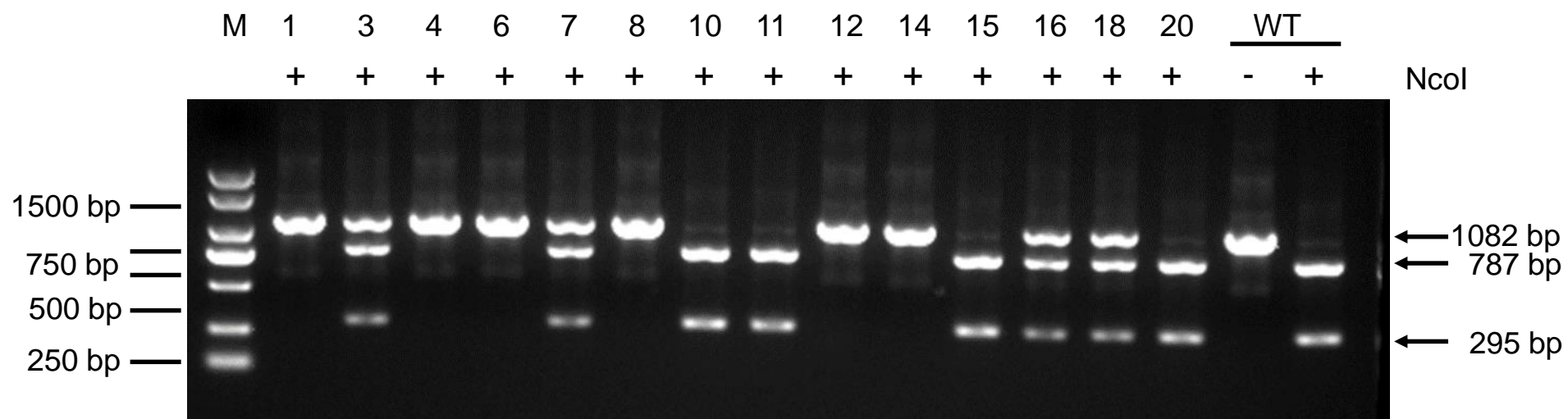

Supplement: Supplementary file 1 [file ijms-24-03071-s001.zip › Fig.S3.pdf]

(a)

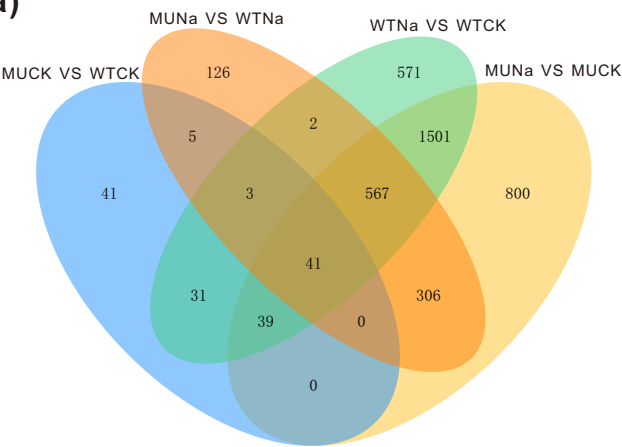

(b)

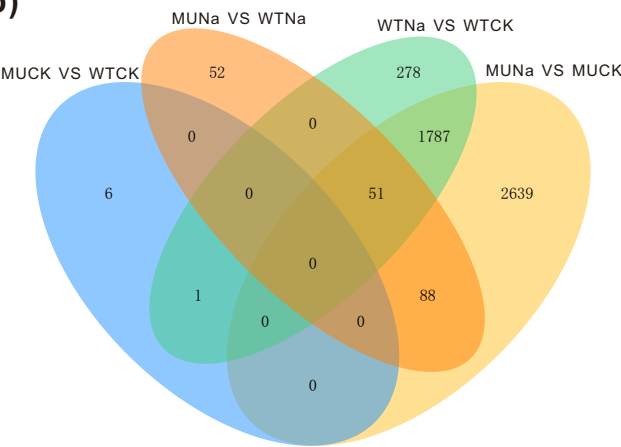

(c)

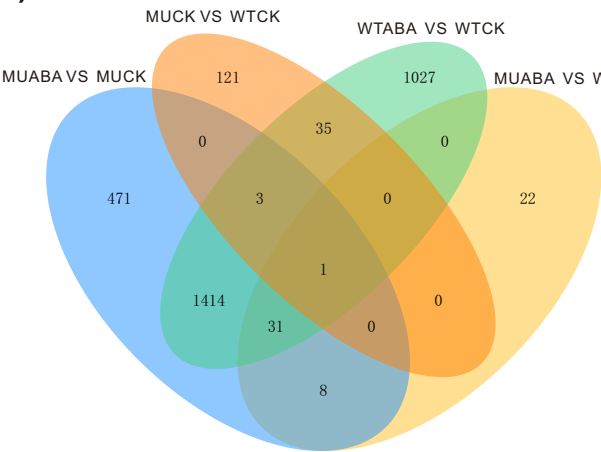

(d)

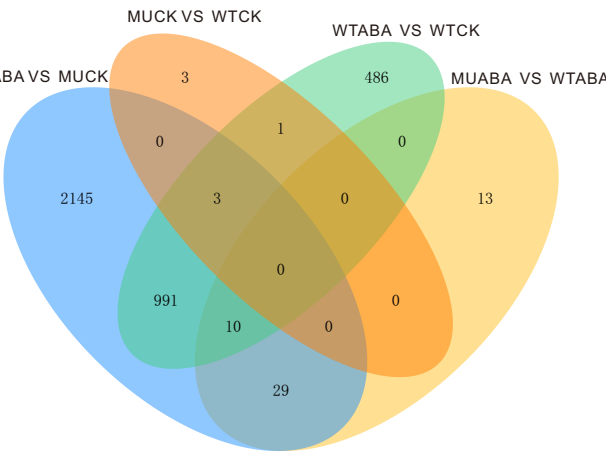

Supplement: Supplementary file 1 [file ijms-24-03071-s001.zip › Fig.S4.pdf]

MUCK\_vs\_WTCK

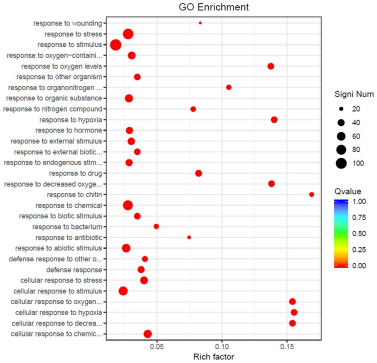

MUNa\_vs\_MUCK

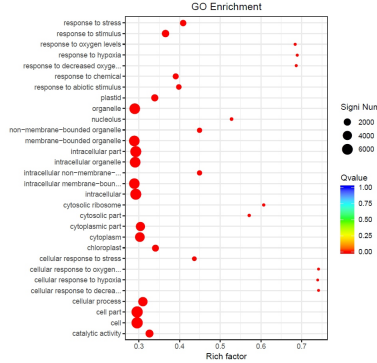

MUNa\_vs\_WTNa

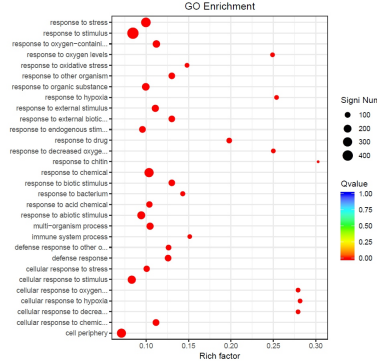

WTNa\_vs\_WTCK

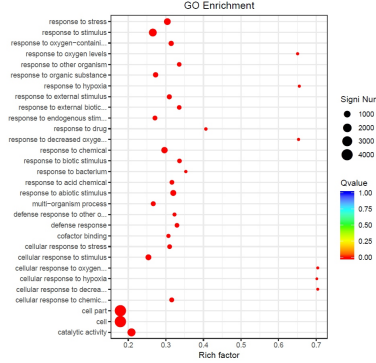

MUABA\_vs\_MUCK

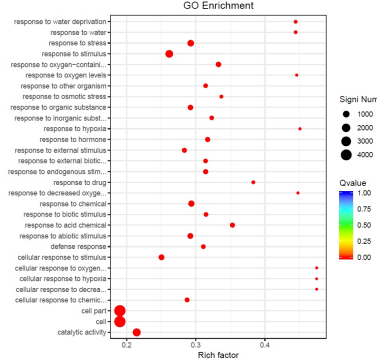

MUABA\_vs\_WTABA

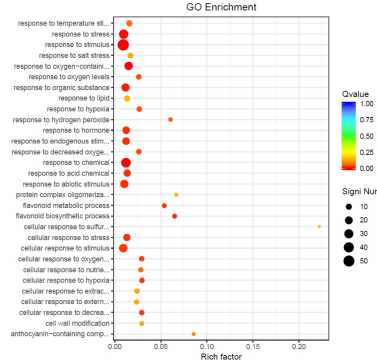

WTABA\_vs\_WTCK

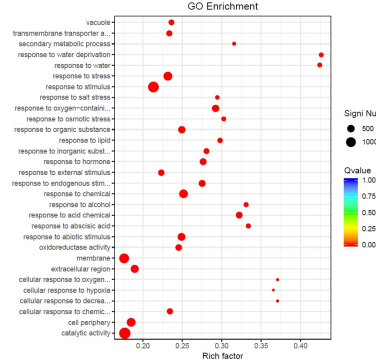

Supplement: Supplementary file 1 [file ijms-24-03071-s001.zip › Fig.S5.pdf]

## MUCK\_vs\_WTCK

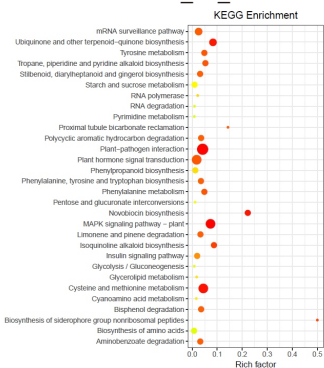

## MUNa\_vs\_MUCK

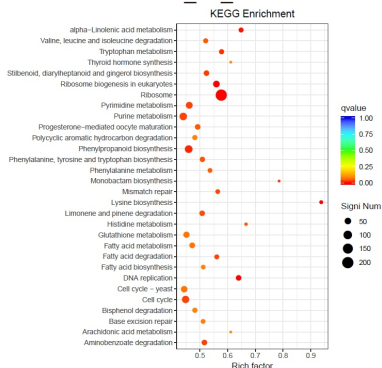

## MUNa\_vs\_WTNa

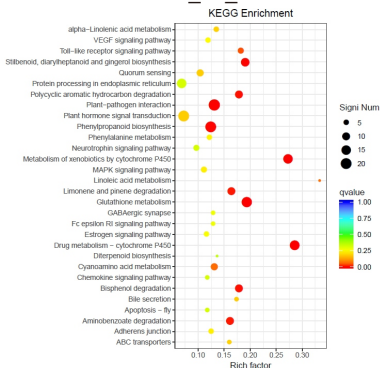

## WTNa\_vs\_WTCK

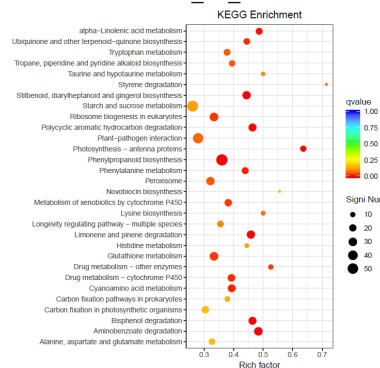

## MUABA\_vs\_MUCK

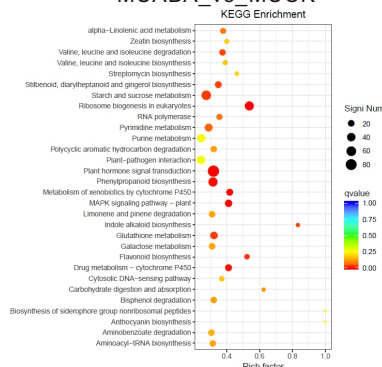

## MUABA\_vs\_WTABA

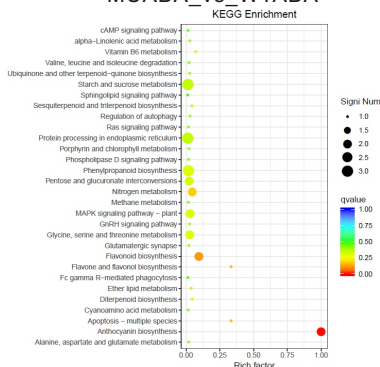

## WTABA\_vs\_WTCK

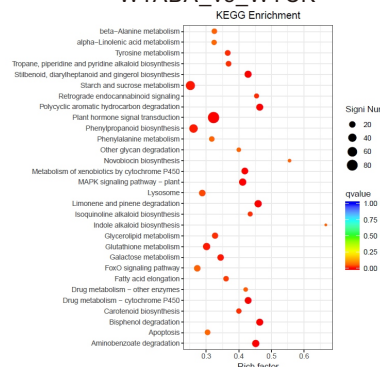

Supplement: Supplementary file 1 [file ijms-24-03071-s001.zip › Fig.S6.pdf]

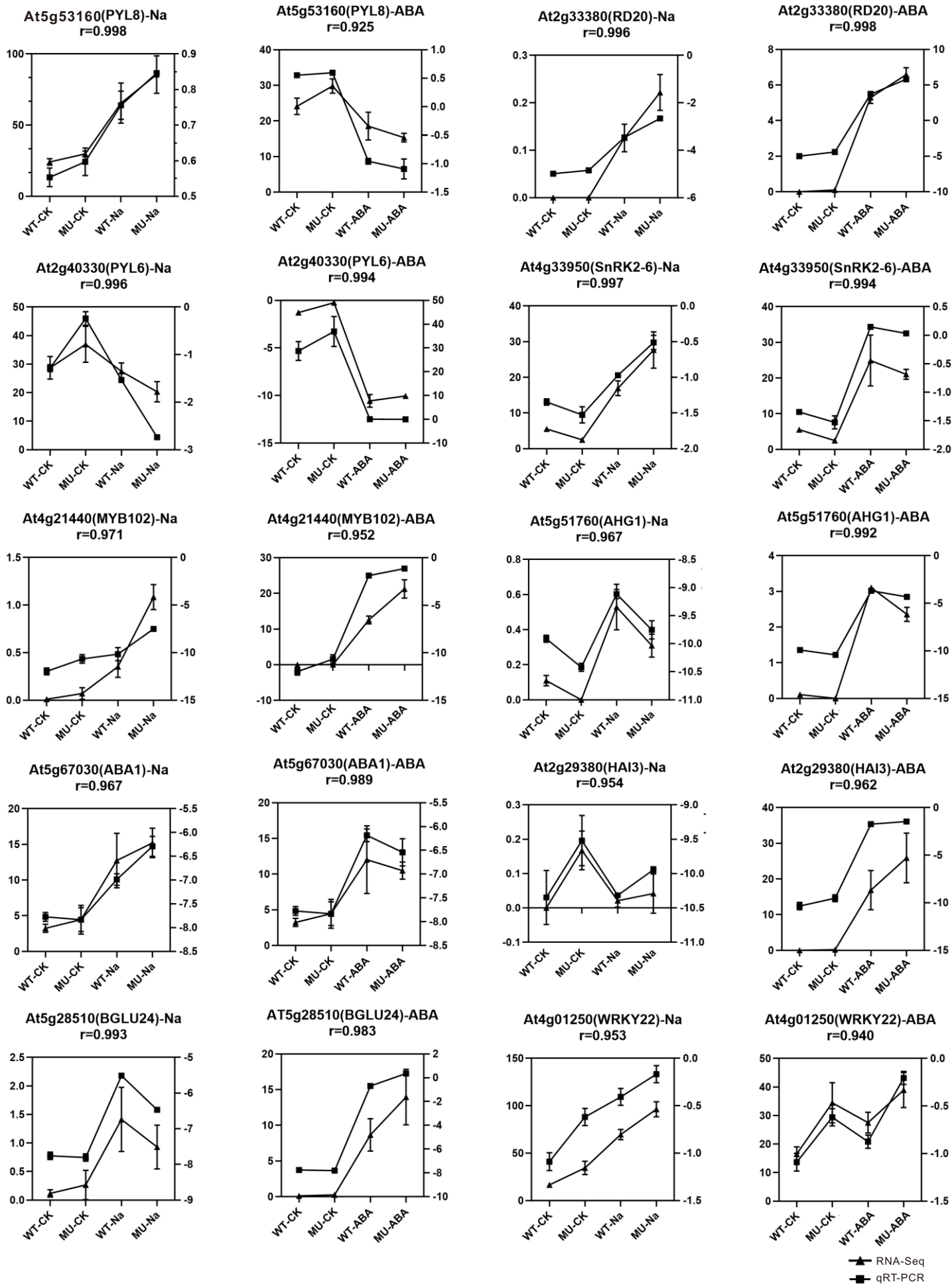

Supplement: Supplementary file 1 [file ijms-24-03071-s001.zip › Fig.S7.pdf]
